# Medical Department of Lind University

**Published:** 1863-10

**Authors:** 


MEDICAL DEPARTMENT OF LIND UNIVERSITY.—A new College edifice is in course of erection for the Medical Department of Lind University, in Chicago, Ill., to be completed in September, and used for the next annual course of instruction. It is of brick, three stories high, and will combine all desirable conveniences and comforts. The name of this University having been changed by the Board of Trustees to that of *Lake Forest University*, a new name for the medical school became necessary, and the Faculty have given to it the title of the *Chicago Medical College*, by which it will hereafter be known.

